# Which is a real valuable screening tool for lung cancer and measure thoracic diseases, chest radiography or low-dose computed tomography?: A review on the current status of Japan and other countries

**DOI:** 10.1097/MD.0000000000038161

**Published:** 2024-05-10

**Authors:** Ikuma Kasuga, Yoshimi Yokoe, Sanae Gamo, Tomoko Sugiyama, Michiyo Tokura, Maiko Noguchi, Mayumi Okayama, Rei Nagakura, Nariko Ohmori, Takayoshi Tsuchiya, Atsushi Sofuni, Takao Itoi, Osamu Ohtsubo

**Affiliations:** a Department of Medicine, Healthcare Center, Shinjuku Oiwake Clinic and Ladies Branch, Seikokai, Tokyo, Japan; b Department of Internal Medicine, Faculty of Medicine, Tokyo Medical University, Tokyo, Japan; c Department of Nursing, Faculty of Human Care, Tohto University, Saitama, Japan; d Department of Gastroenterology and Hepatology, Tokyo Medical University, Tokyo, Japan; e Department of Clinical Oncology, Tokyo Medical University, Tokyo Japan; f Department of Medicine, Kenkoigaku Association, Tokyo Japan.

**Keywords:** cardiovascular disease, chronic obstructive pulmonary disease, computed tomography, lung cancer, pulmonary infection, radiography, screening

## Abstract

Chest radiography (CR) has been used as a screening tool for lung cancer and the use of low-dose computed tomography (LDCT) is not recommended in Japan. We need to reconsider whether CR really contributes to the early detection of lung cancer. In addition, we have not well discussed about other major thoracic disease detection by CR and LDCT compared with lung cancer despite of its high frequency. We review the usefulness of CR and LDCT as veridical screening tools for lung cancer and other thoracic diseases. In the case of lung cancer, many studies showed that LDCT has capability of early detection and improving outcomes compared with CR. Recent large randomized trial also supports former results. In the case of chronic obstructive pulmonary disease (COPD), LDCT contributes to early detection and leads to the implementation of smoking cessation treatments. In the case of pulmonary infections, LDCT can reveal tiny inflammatory changes that are not observed on CR, though many of these cases improve spontaneously. Therefore, LDCT screening for pulmonary infections may be less useful. CR screening is more suitable for the detection of pulmonary infections. In the case of cardiovascular disease (CVD), CR may be a better screening tool for detecting cardiomegaly, whereas LDCT may be a more useful tool for detecting vascular changes. Therefore, the current status of thoracic disease screening is that LDCT may be a better screening tool for detecting lung cancer, COPD, and vascular changes. CR may be a suitable screening tool for pulmonary infections and cardiomegaly.

## 1. Introduction

Japan has its own medical checkup system, and most of the people on working-age generation undergo a systemic medical examination annually. A combination of chest radiography (CR) and sputum cytology is routinely recommended for initial assessment for lung cancer screening in our country and mainly used as a common imaging modality for every year screening.^[1]^ It is widely available, involves less radiation exposure, and is less expensive than computed tomography (CT). Moreover, the recently developed automated analysis of CR can help in population screening during health checkups. Furthermore, it can assist pulmonologists and radiologists in interpreting and triaging, thereby easing their workloads.^[2–4]^ However, there is a problem in CR screening for lung cancer that we cannot overlook. CR has not a high sensitivity and is usually considered an ineffective screening modality for the detection of lung nodules that are <10 mm.^[5,6]^ Hence, lung cancers detected using this method are often in an advanced stage at the time of diagnosis.^[7]^ In fact, our previous study demonstrated that CR did not contribute to the early detection of lung cancer at health checkups.^[8]^ In addition, a recent randomized controlled trial on lung cancer screening by CR did not show a reduction in mortality compared with that following usual care.^[9]^ This means that CR may not contribute to the early detection of lung cancer and may not be a useful modality for lung cancer screening.

In contrast, recent studies showed the utility of low-dose computed tomography (LDCT) in the early detection and early resection of lung cancer and other thoracic malignancy.^[10,11]^ Recent large randomized trials also showed the efficacy of LDCT in lung cancer screening which contributed to early detection and saved lives compared with screening using CR.^[12]^ Therefore, we should reconsider that CR screening which recommended in Japan is a valuable method or not for lung cancer.

On the other hand, CR and LDCT should also be considered important screening tools for other thoracic diseases such as chronic obstructive pulmonary disease (COPD), pulmonary infectious diseases, and cardiovascular disease (CVD) because they are more frequently identified than lung cancer, during health checkups.^[10]^ Although CR and LDCT have been discussed about mainly in the detection of lung cancer, their utility for screening other thoracic diseases has not been well discussed to date.

Therefore, we herein review the current status of CR and LDCT as veridical screening tools for not only lung cancer but also for other major thoracic diseases as well.

## 2. Lung cancer screening

**Figure 1.**
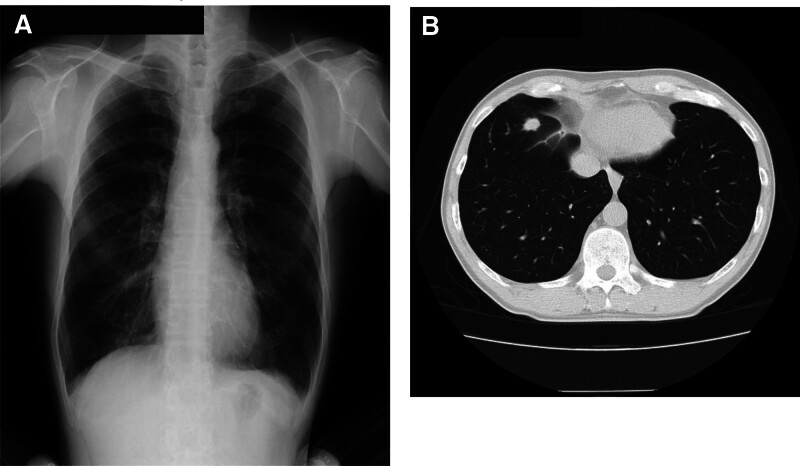
CR and LDCT images of lung cancer case taken on the same day. (A) CR reveals no abnormal opacity. (B) LDCT shows a tumorous lesion in the right lower lung field. This patient was diagnosed with stage I lung cancer. CR = chest radiography, LDCT = low-dose computed tomography.

Lung cancer is the leading cause of cancer-related deaths worldwide because of its heterogeneity, aggressive nature, and poor prognosis, despite its lower incidence compared with that of other major cancers, such as gastric, colorectal, and prostate cancer in men and breast cancer in women.^[13–16]^ Therefore, detection of lung cancer at an early stage during health checkups is important for prompt surgical resection and reduction of cancer-related deaths. The use of CR and LDCT as screening tools for lung cancer has long been debated. In Japan, CR and sputum cytology are recommended for lung cancer screening over the age of 40 years old by the Ministry of Health, Labor and Welfare, and LDCT is not recommended for lung cancer screening. Therefore, CR is basically administered as an initial assessment of lung cancer screening everywhere in Japan. LDCT screening for lung cancer is currently administered in limited local government to date. Several studies in other countries have demonstrated that screening using LDCT contributes to early-stage detection of lung cancer and improves outcomes when compared to screening using CR (Fig. 1A and B).^[17–19]^ A recent study by the National Lung Screening Trial reported in 2011 that adhered to a protocol of annual LDCT lung cancer screening and follow-up showed reduced lung cancer mortality by 20% compared with that following CR screening in smokers.^[12]^ This study is meaningful because this is the first large randomized trial to demonstrate the efficacy of CT in lung cancer screening. Recently, new other reports have also shown similar results of the former randomized trial and demonstrated that the utility of LDCT in the early detection and resection of lung cancer and other thoracic malignancy.^[10,20–22]^ These results obtained that LDCT screening may be a better method for lung cancer screening than CR screening.

However, another recent population-based study identified a key problem in implementing LDCT screening in everyday practice as used in trial settings.^[23]^ The prevalence of CT screening among eligible people in the United States (US) has remained virtually stagnant over the past few years: 3.3% in 2010 and 3.9% in 2015. Moreover, the majority of cigarette smokers continue to undergo screening with CR rather than LDCT.^[23,24]^ The acceptance rate of LDCT for lung cancer screening is much lower than that of other disease screening tests; for example, in many countries, a majority of eligible women attend screening mammography.^[25]^ The primary reason for the low prevalence of CT screening is its high cost. LDCT screening costs approximately US$ 100 per test and is much more expensive than CR screening. Another problem that should be discussed is that CT imaging involves more radiation exposure than CR imaging. The average dose for conventional standard chest CT is approximately 7 to 8 millisievert (mSv), resulting in a high exposure to radiation.^[26]^ The average dose for LDCT imaging is approximately 1.5 mSv, which is half of the natural ambient exposure dose of approximately 3 mSv per year.^[27]^ In addition, the average dose of radiation exposure with LDCT is less than that for upper gastrointestinal series (UGI), which is performed every year during health checkups in Japan.^[28]^ Even if LDCT screening is repeated every 3 months, radiation exposure per year is less than with standard CT. Therefore, LDCT may not result in an undue increase in radiation exposure.^[29]^ Currently, in Japan, UGI and mammography are performed for gastric and breast cancer screening, respectively; however, LDCT has not been recommended for lung cancer screening. It should also be noted that LDCT requires less time, is less uncomfortable than UGI, and is less painful than mammography.

**Figure 2.**
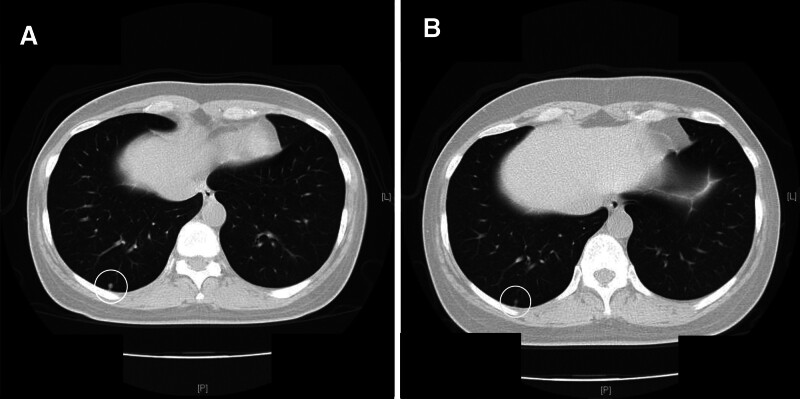
LDCT image of a case taken for 1-yr period. (A) LDCT showing a small nodule located in the right lower lung field with a slight pleural indentation (circled) at first examination. (B) LDCT of this case 1 yr after the first examination. The nodular shadow shows rather improvement (circled) compared with that at the first examination. LDCT = low-dose computed tomography.

Another important aspect to consider when evaluating the usability of CR and LDCT as cancer screening procedures is that there were false-positive scans for no cancer-related nodules. The false-positive findings often occur for benign tumorous lesions, especially during LDCT screening.^[30,31]^ These cases are followed up periodically because the pulmonary nodules are only small in size (Fig. 2A and B) and patients were often distressed during the follow-up period.

Thus, current status of lung cancer screening is that LDCT can provide evidence of possibly early-stage detection compared with CR, these make it difficult for it to become widely used.

## 3. COPD screening

**Figure 3.**
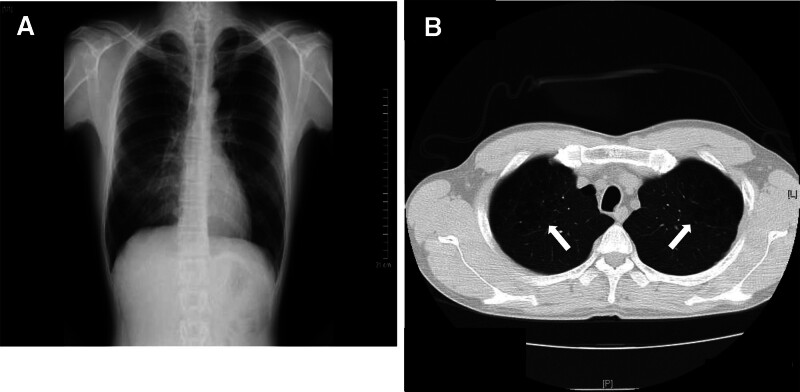
CR and LDCT images of COPD case taken on the same day. (A) CR reveals no abnormal opacity. (B) LDCT shows a number of low-attenuation areas in the bilateral upper lung fields (white arrows). COPD = chronic obstructive pulmonary disease, CR = chest radiography, LDCT = low-dose computed tomography.

COPD has become a major cause of morbidity and mortality worldwide; it also has a high incidence.^[32,33]^ Unfortunately, COPD is not well recognized in Japan despite its estimated prevalence of 5.3 million.^[34]^ For the most part, the diagnosis of COPD is based on the presence of clinical symptoms and the ratio of postbronchodilator forced expiratory volume in 1 second to forced expiratory vital capacity using a spirometer being <70%. Diagnostic imaging using CR and CT plays a subsidiary role in the detection of COPD. CR shows some distinctive features of COPD such as radiolucency of the lung field, depression and flattening of the diaphragm, a tear-drop heart, and the absence of vasculature, but these findings are recognized mainly in moderate and severe phases of the disease.^[35]^ Individuals who show these CR findings usually have some clinical symptoms and visit a hospital for further examination. LDCT scanning is more sensitive in detecting early diagnostic signs of COPD, which are low-attenuation areas in the lung field, while no such findings are seen on CR imaging (Fig. 3A and B).^[36,37]^ There are high expectations regarding early detection and implementation of smoking cessation treatments for COPD resulting from LDCT screening.^[10]^ Cigarette smoking is the most commonly encountered and readily identifiable risk factor for COPD, and the early initiation of smoking cessation is important to prevent the exacerbation of COPD.^[38–43]^ However, in Japan a major problem is that COPD and its clinical importance have not been well recognized among people. Therefore, majority of smokers still undergo CR screening every year and it has led to a delay in implementing smoking cessation treatments.

## 4. Pulmonary infections screening

**Figure 4.**
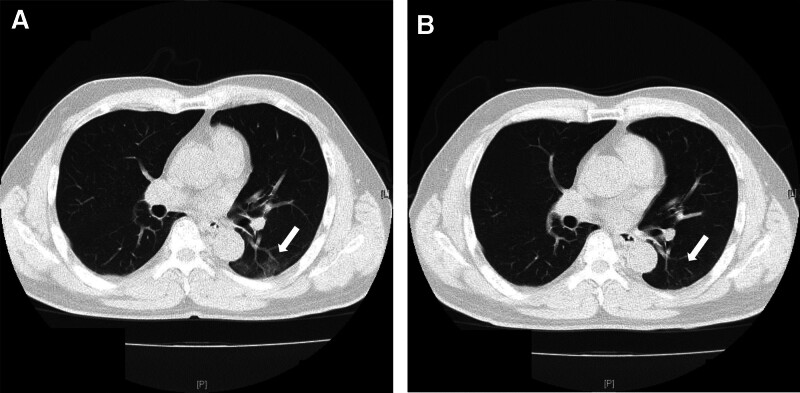
LDCT image of pulmonary infection case taken for 1-yr period. (A)LDCT showing inflammatory change in the left lower lung field (white arrow). (B)LDCT of this case 1 yr after the first examination. This lesion finally improved spontaneously without any medication (white arrow). LDCT = low-dose computed tomography.

The most commonly encountered abnormal lesion among the working-age population is pulmonary infections, whereas lung cancer and COPD are usually detected in older individuals.^[10,44]^ The most frequently identified cause of pulmonary infection is community-acquired pneumonia caused by *Streptococcus pneumoniae, Staphylococcus aureus*, or *Mycoplasma*; the condition usually improves spontaneously.^[45]^ The recently prevalent SARS-CoV-2 often caused pneumonia; however, after the new omicron outbreak, these cases have now reduced.^[46–49]^ A few specific infections require treatment with antibiotics, such as tuberculosis or pneumomycosis. Although LDCT can reveal small inflammatory changes that are not observed in CR, many of these cases improved spontaneously (Fig. 4A and B).^[10]^ Therefore, LDCT screening for pulmonary infections may be less useful than that for lung cancer or COPD. CR screening may be sufficient and suitable for the detection of pulmonary infections, especially during health checkups, as has been previously reported.^[50–52]^

## 5. CVD screening

CR and LDCT should also be important screening tools for CVD. Among CVD, congenital heart disease (CHD) is occasionally found in the younger generation during screening. This is because some cases of CHD are subclinical CHD and remain undiagnosed until adulthood.^[53]^ There is a report that recent advances in 3-dimensional CT have improved the spatial resolution, allowing for a detailed evaluation of cardiac morphology; however, the utility of LDCT in the detection of CHD remains unclear.^[53]^ A previous study showed that CR could detect more cases of cardiomegaly, an incipient sign of CHD and cardiomyopathy, than LDCT.^[10]^ CR is nearly always obtained in the management of suspected CVD and provides an important means to appreciate the morphological changes in the heart, such as cardiomegaly, in cases of CHD.^[54,55]^ However, vascular changes in the aorta and coronary artery, such as sclerosis, dilatation, and dissection, are usually found in the middle or older age than in the CHD group. Although it has been reported that aortic calcification detected on CR is closely associated with predictive variables of future CVD, other studies have shown that LDCT is a more effective tool to detect aortic sclerosis and dissection with higher accuracy than CR in screening.^[56–58]^ Moreover, recent studies have shown that aortic CT with ultra-low-dose contrast medium can also maintain high image quality as well as that of conventional dose.^[59,60]^ These make it easier to use as a screening tool for vascular disease. Therefore, LDCT screening is more useful for detecting vascular disease. These results suggest that CR is convenient and adequate tool for detecting cardiomegaly, especially in younger age, whereas, LDCT is a more effective tool for detecting vascular changes, especially in the middle and older age. Therefore, CR and LDCT have own advantage for CVD screening, respectively.

## 6. Conclusion

Although LDCT is not recommended for lung cancer screening in Japan, it may be a better screening tool for detecting lung cancer. It may also useful for the early detection of COPD and vascular changes as well. However, it is difficult for it to become widely used to date owing to its high cost, radiation exposure, and false-positive findings. On the other hand, CR may be a suitable screening tool for pulmonary infections and cardiomegaly compared with LDCT.

## Acknowledgments

The authors would like to thank all the staff of Seikokai Group and Medical four clover Co., Ltd. for their providing technical assistance for this review manuscript. The authors would also like to thank Dr Keisuke Toyama, Professor Emeritus of Tokyo Medical University, for his valuable advice. A summary of this article was presented at a virtual event of Euro-Global Summit on Advances in Clinical and Cellular Immunology held on September 12, 2023. The preliminary data of this article was presented at the Joint Meeting of the 27th International Health Evaluation and Promotion Association and the 4th World Congress on Ningen Dock held from November 26 to December 11, 2020, via live and on-demand web streaming, and at the 19th World Congress on Lung Cancer in Toronto, ON, Canada on September 24, 2018, respectively.

## Author contributions

**Data curation:** Ikuma Kasuga.

**Investigation:** Ikuma Kasuga, Yoshimi Yokoe, Sanae Gamo, Tomoko Sugiyama, Michiyo Tokura, Mayumi Okayama, Rei Nagakura, Nariko Ohmori, Takayoshi Tsuchiya, Atsusi Sofuni, Takao Itoi.

**Methodology:** Ikuma Kasuga.

**Project administration:** Ikuma Kasuga.

**Resources:** Osamu Ohtsubo.

**Supervision:** Maiko Noguchi.

**Writing – original draft:** Ikuma Kasuga.

**Writing – review & editing:** Ikuma Kasuga.
